# Transfersomes: A Novel Vesicular Carrier for Enhanced Transdermal Delivery of Sertraline: Development, Characterization, and Performance Evaluation

**DOI:** 10.3797/scipharm.1208-02

**Published:** 2012-08-31

**Authors:** Ankit Gupta, Geeta Aggarwal, Samita Singla, Ritika Arora

**Affiliations:** 1 Department of Pharmaceutics, Rayat Institute of Pharmacy, Railmajra, Ropar, Punjab – 140104, India.; 2 Department of Pharmaceutics, Rayat and Bahra Institute of Pharmacy, Sahauran, Distt. Mohali, Punjab – 140104, India.

**Keywords:** Sertraline, Transfersomes, Transdermal, Permeation studies, *In vivo* study

## Abstract

The aim of the present study was to investigate transfersomes as a transdermal delivery system for the poorly soluble drug, sertraline, in order to overcome the troubles associated with its oral delivery. Different transfersomal formulations were prepared with non-ionic surfactant (span 80), soya lecithin, and carbopol 940 by the rotary evaporation sonication method. The prepared formulations were characterized for light microscopy, particle size analysis, drug entrapment, turbidity, drug content, rheological studies, *in vitro* release, *ex vivo* permeation, and stability studies. The optimized formulation was evaluated for *in vivo* studies using the modified forced swim model test. FTIR studies showed compatibility of the drug with excipients. The result revealed that sertraline in all of the formulations was successfully entrapped with uniform drug content. Transfersomal gel containing 1.6% of the drug and 20% of span 80 was concluded to be the optimized formulation (EL-SP4), as it showed maximum drug entrapment (90.4±0.15%) and cumulative percent drug release(73.8%). The *ex vivo* permeation profile of EL-SP4 was compared with the transfersomal suspension, control gel, and drug solution. The transfersomal gel showed a significantly higher (p<0.05) cumulative amount of drug permeation and flux along with lower lag time than the drug solution and drug gel. It also owed to better applicability due to the higher viscosity imparted by the gel rather than the transfersomal suspension, and no skin irritation was observed. The modified forced swim test in mice revealed that the transfersomal gel had better antidepressant activity as compared to the control gel. Thus, the study substantiated that the transfersomal gel can be used as a feasible alternative to the conventional formulations of sertraline with advanced permeation characteristics for transdermal application.

## Introduction

Depression is one of the most common psychiatric disorders and is characterized by feelings of intense sadness, helplessness, worthlessness, and impaired functioning [1]. Sertraline, an antidepressant drug, is a selective serotonin reuptake inhibitor (SSRI) administered orally alone or in combination with hydrochloride in a daily oral dose of 50 mg. But various problems are associated with its oral delivery such as extensive first-pass metabolism, gastrointestinal disturbances such as nausea, dry mouth, diarrhea, decreased appetite *etc*., and ultimately its poor bioavailability (40–45%), which required this drug to be taken in high doses in order to maintain adequate plasma levels [2].

Transdermal delivery of sertraline is a better-suited alternative to surmount the problems associated with its oral delivery. The transdermal route, besides being convenient and safe, offers several advantages over conventional ones, such as avoidance of GI incompatibility, variable GI absorption, avoidance of first-pass metabolism, improved bioavailability, reduced frequency of administration, improved patient compliance, and rapid termination of drug input [3].

Despite decades of research, the barrier function of the stratum corneum still remains a problem, which makes the development of new transdermal drug delivery systems an interesting challenge. Vesicular systems have been widely explored as surrogate vehicles for topical and transdermal drug delivery. Their benefits in enhancing drug permeation have been well established [4]. Despite the strong rationale for the use of vesicles in transdermal drug delivery, the major problem in the development of vesicular systems at industrial and clinical levels is their poor stability.

Different approaches have been proposed to enhance the stability of the vesicular system. Transfersomes (transfersomes) offer a versatile delivery concept for improving the stability as well as the potential to be used with a wide range of active compounds. They are (quasi) metastable, which makes the vesicle membrane ultra-flexible, and thus, the vesicles are highly deformable that squeeze through pores in the stratum corneum less than one-tenth of their own diameter when applied under non-occlusive conditions. Thus, even sizes up to 200–300 nm can penetrate intact skin. It is primarily due to the remarkable strong membrane adaptability that allows the transfersomal vesicles to lodge in a confining pore, and thus permeate that pore [3, 5, 6]. But the low viscosity of transfersomal suspensions constrains its application in transdermal delivery due to its cumbersome use. Biocompatible gels having weak interactions with surfactants have already been explored to modify the rheological behavior of the transfersomal suspension. Thus, the incorporation of the transfersomal suspension into the gel matrix can result in a transfersomal gel which may be more relevant for transdermal application.

This study is aimed to incorporate sertraline in the transfersomal gel system for transdermal administration to avoid problems associated with its oral delivery, and to enhance the permeation of the drug through the skin and ultimately enhance the bioavailability. The prepared system was optimized and evaluated for *in vitro* release studies. An *ex vivo* permeation study was also conducted to assess the transdermal ability of this system to deliver sertraline. Furthermore, skin irritation studies and an *in vivo* study of the optimized transfersomal formulation was also conducted using the modified forced swim model test in mice.

## Materials and Methods

### Materials

Sertraline was received as a gift sample from Medisys Biotech Ltd, India. Span 80 and soya lecithin were obtained from S.D. Fine chemicals Ltd., India. All other chemicals used in this study were of analytical grade. Swiss white male/female mice (25–30 g), Wistar rats (150–250 g), and a guinea pig (700–1000 g) were obtained from the Central Animal House facility of the Rayat Institute of Pharmacy, Railmajra, Punjab, India. The experimental protocols were approved by the Institutional Animal Ethics Committee (IAEC) as per guidelines of the Committee for the Purpose of Control and Supervision of Experiments on Animals (CPCSEA), Government of India.

### Formulation of sertraline transfersomes

The transfersomes were formulated by the conventional rotary evaporation sonication method [7, 8]. Transfersomes containing phospholipids (soya lecithin), surfactant (span 80), and the drug (sertraline) were formulated. The drug concentration varied from 0.5 to 1.8 wt. % and the surfactant concentration varied from 5 to 25 wt % of the phospholipids. The phospholipids and surfactant were taken in a clean, dry, round-bottom flask and this lipid mixture was dissolved in a small quantity of ethanol. The organic solvent was removed by rotary evaporation under reduced pressure at 40°C. Final traces of solvents were removed under vacuum overnight. The deposited lipid film was hydrated with 7 % v/v ethanol *i.e.* solution of the drug by rotation at 60 rpm for 1 h. The resulting vesicles were kept for 2 h at ambient temperature to swell and form into large multilamellar vesicles. These were sonicated in a bath for 10 min to achieve smaller vesicles.

The varied amounts of the drug were added (0.5 to 1.8 %) to determine the drug-loading capacity of transfersomes. All of the drug-loaded vesicular formulations were examined for maximum entrapment efficiency and for the appearance of drug crystals over a period of 14 days using an optical microscope.

### Drug excipient interaction study

Fourier Transform Infrared Spectroscopy (FTIR) of the pure drug sertraline and a mixture of the drug (100 mg) with excipients (100 mg of span 80 and soya lecithin) was taken using the Perkin Elmer FTIR spectrophotometer (RXIFT-IR system, USA). The sample was prepared with potassium bromide and data were collected over a spectral range of 450−4000 cm^−1^. Also, the UV spectra of the pure drug and drug with excipients were also observed.

### Characterization of elastic liposomal formulations

#### Entrapment efficiency

Percentage entrapment efficiency was conducted by the centrifuge method. 100 mg of the transfersomal formulation was weighed and dispersed in 10 mL of phosphate buffer saline (PBS) pH 6.8. The transfersomal dispersion obtained was centrifuged (REMI LJ 01, Mumbai, India) at 10000 rpm for 40 min. The clear fraction (supernatant) was used for the determination of free drug. The drug concentration in the resulting solution was assayed by a UV spectrophotometer (Shimadzu- 1700, Japan) at 274 nm [9].

The percentage of drug encapsulation was calculated by the following equation:

$$\text{Entrapment}\;\text{efficiency}\;\left(\%\right)=\left[\left({\text{C}}_{\text{t}}-{\text{C}}_{\text{f}}\right)/{\text{C}}_{\text{t}}\right]\times 100$$

Where C_t_ is the concentration of total drug and C_f_ is the concentration of unentrapped drug.

#### Morphology and Structure of transfersomes

The morphology and structure of the drug-loaded transfersomes were determined with the aid of Transmission Electron Microscopy (TEM): (Hitachi H7500, Japan). Transfersomal formulations were diluted with water. A drop of the diluted suspension was then directly deposited on the holey film grid, stained by 1% aqueous solution of phosphotungestic acid, and observed after drying.

Vesicles without sonication were also visualized by using an optical microscope (Leica digital microscope, Germany). A thin film of transfersomes was spread on a slide and a cover slip was placed over it and then observed under the optical microscope [10].

#### Vesicle size and size distribution

Analysis of the transfersome vesicle size before sonication was determined by optical microscopy using a stage eyepiece micrometer calibrated using a micrometer scale. The Polydispersity Index (PDI) measurement was carried out by dynamic light scattering with the Zetasizer HSA 3000 (Malvern Instruments Ltd, UK). All samples were subjected to sonication prior to PDI determination [11].

#### Turbidity measurements

The transfersomes were diluted with distilled water to give a total lipid concentration of 0.312 mm. After rapid mixing by sonication for 5 min, the turbidity was measured as the absorbance at 274 nm with a UV- visible spectrophotometer (Shimadzu-1700, Japan).

#### Development of a secondary topical vehicle

All of the formulations (EL-SP1 to EL-SP5) were found to be in the nano size range and were therefore incorporated into the gel matrix resulting in transfersomal gel. Carbopol 940 was selected as the gel matrix base. Carbopol 940 was swelled in a small amount of water for 24 h and a high viscous solution was obtained, and the transfersomal suspension was slowly added to the viscous solution of carbopol 940 under magnetic stirring. The concentration of carbopol 940 in the transfersomal gel was 1% (w/w). The pH values were subsequently regulated to 6–9 by using triethanolamine.

#### Drug content determination

The amount of drug contained in the transfersomal gel was determined by dissolving 100 mg of the formulation in 10 mL of ethanol. The mixture was analysed by a UV-Visible spectrophotometer at 274 nm against ethanol as a blank [12].

#### Viscosity measurement and rheological behavior of transfersomal gel

The viscosity of the prepared formulations was determined at different angular velocities at 32.0 ± 0.1°C using spindle no. 4 (Brookfield DV-II+ pro viscometer) [13]. The transfersomal gel formulations were evaluated for their rheological behavior using cone and plate configuration (40 mm cone with 2.5 deg cone angle). Rheology studies were conducted in the shear rate range of 50.63–250.7 s^−1^ at 25°C. The consistency index and flow index were calculated from the Power law equation:

$$\tau ={\text{Kr}}^{\text{n}}$$

Where, τ = shear stress, r = shear rate, K = consistency index, n = flow index

Taking log on both sides,

Logτ=Log K+n logr

Thus from the plot of the log of shear stress versus the log of shear rate, the slope of the plot was taken as the flow index and the Y-intercept gave the consistency index.

#### In vitro drug release studies through cellophane membrane

The *in vitro* permeation behaviour of sertraline from all transfersomal gel formulations and the control gel formulation (containing drug, lecithin, and span 80) were investigated using cellophane membrane (Molecular weight cut of 12000–14000, HI Media Ltd, Mumbai, India). The vertical type of the Franz Diffusion cell was designed, fabricated, and validated prior to the permeation study. The cellophane membrane was mounted on a diffusion cell assembly with an effective diffusion area of 2.303 cm^2^. The receptor compartment consisted of a 22.5 mL phosphate buffer at pH 6.8 as the receptor fluid agitated at 100 rpm, and was maintained at 37 ± 0.5°C throughout the experiments. The prepared formulation was applied to the membrane in the donor compartment. An aliquot of 2 mL sample was withdrawn at suitable time intervals and replaced immediately with an equal volume of fresh diffusion medium. The cumulative amount that permeated across the cellophane membrane was calculated and plotted against time.

#### Release kinetics

To study the release kinetics, data obtained from *in vitro* permeation studies were fitted in various kinetic models: zero order as the cumulative percent of drug permeated *vs.* time, first order as the log cumulative percentage of drug remaining *vs.* Time, and Higuchi’s model as the cumulative percent drug permeated *vs.* square root of time. To determine the mechanism of drug release, the data were fitted into the Korsmeyer- Peppas model as the log cumulative percentage of drug released *vs.* log time, and the exponent n was calculated from the slope of the straight line. For the slab matrix, if the exponent is 0.5, then the diffusion mechanism is fickian; if 0.5<n <1.0, the mechanism is non- fickian, n = 1 to Case II (relaxational) transport, and n > 1 to super case II transport.

### Ex vivo drug permeation using a diffusion cell

#### Preparation of Wistar rat skin for permeation studies

Hairless animal skin was used for the permeation studies. Hair on the dorsal skin of the sacrificed animal was removed with an animal hair clipper, subcutaneous tissue was surgically removed, and the dermis side was wiped with isopropyl alcohol to remove residual adhering fat. The skin was washed with PBS pH 6.8. The prepared skin was wrapped in aluminium foil and stored in a deep freezer at −20°C for further use. The skin was defrosted at room temperature when required.

#### Skin permeation studies

The selected formulations on the basis of entrapment efficiency, drug content, and permeation through the cellophane membrane were subjected to permeation studies through rat skin using the Franz Diffusion cell in a similar manner as through the cellophane membrane. The cumulative amount of drug permeated was compared with the transfersomal suspension, control gel, and drug solution. The cumulative amount of drug permeated across the rat skin was plotted against time and the flux was calculated as drug permeated per cm^2^ per hour.

#### Skin irritation studies

Skin irritation studies were carried out according to the Ammar technique. Four groups of guinea pigs with three animals each of either sex were used to study the hypersensitivity reaction on the skin. The first group of animals served as the control group *i.e.* no drug, the second group was applied with transfersomal gel (dose equivalent 10 mg/kg), the third group was applied with the control gel, and the last group was applied with drug solution every day up to 7 days, and finally, the application sites were graded. The erythema and edema were scored as follows: 0 for none, 1 for slight, 2 for well defined, 3 for moderate, and 4 for scar formation and severe erythema and edema [15].

#### In vivo studies using the modified forced swim model test

Three groups of Swiss mice (each group containing three mice) were individually forced to swim inside vertical Plexiglas cylinders containing water maintained at 25 ± 2°C. The behavioural cylinder was 60 cm high and 20 cm wide maintained at 25°C, filled with 30 cm of water, so that the mice could not support themselves by touching the bottom with their paws or tail. The first group served as the control group, *i.e* without the drug, the second group was applied with the control gel (PG) (drug dose equivalent to 10mg/kg), and the third group was treated with transfersomal gel (TG) (equivalent dose as in group 2). The FST included two phases: an initial 15min pretest followed by a 5min test 24 h later. After each session, the mice were removed from the cylinders, dried with towels, and placed into heated cages for 10min, and then returned to their home cages. The total duration of immobility, struggling, and swimming were measured during a 5 min test. A mouse was judged to be immobile whenever it remained passively floating in the water in a slightly hunched but upright position, its head just above the surface. Struggling was considered when the mice made active movements with their forepaws in and out of the water along the side of the swim chamber, whereas swimming was considered when the mice made active swimming or circular movements [16].

### Stability Studies

Stability of the product may be defined as the capability of a particular formulation to remain within the physical, chemical, therapeutic, and toxicological specifications. The optimized formulation was stored in glass vials at room temperature and then kept in a refrigerator (4–8°C) for 3 months. Parameters like morpholgy, drug leakage, and drug entrapment were evaluated at pre-determined time intervals i.e. at 0, 15, 30, 45, 60, 75, 90 days.

### Statistical analysis

All of the studies were carried out in triplicates. The statistical analysis was performed using Student’s unpaired *t*-test. The steady-state flux was determined from the slope of the linear portion of the cumulative amount permeated versus time plot. The lag time (T_lag_) was determined by extrapolating the linear portion of the cumulative amount permeated versus time curve to the abscissa. The enhancement ratio of the flux (E_pen_) was calculated as:

$${\text{E}}_{\text{pen}}={\text{P}}_{\text{treatment}}/{\text{P}}_{\text{control}}$$

Where P_treatment_ is the flux of formulation and P_control_ is the flux of the control group.

## Results and discussion

### Formulation of transfersomes

Span 80 was selected as the edge activator surfactant for the transfersomal formulation as it is biocompatible and pharmaceutically acceptable [17]. Phospholipid was used as the bilayer-forming agent and ethanol was used as the hydrating agent because ethanol is known to extract stratum corneum lipids and alter the barrier property of the intracellular lipoidal route, thereby allowing higher drug permeation [14].

FTIR data of the pure drug sertraline and the drug with excipients (span 80 and soya lecithin) suggested that there was no interaction between the drug and the excipients used.

### Effect of span 80 concentration on entrapment efficiency

**Fig. 1.**
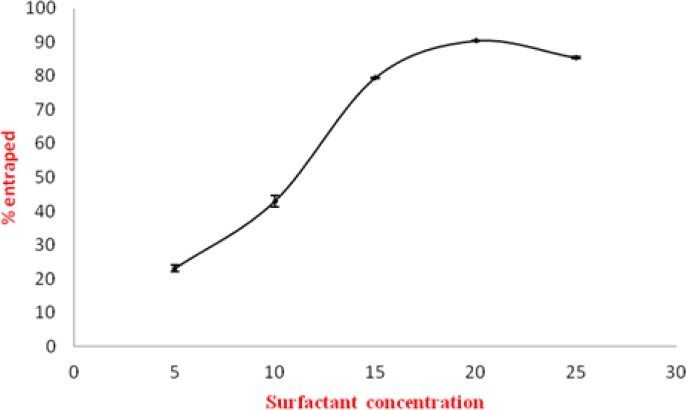
Effect of surfactant concentration on entrapment efficiency of sertraline

To optimize the concentration of span 80 in the vesicles, the entrapment efficiency of various transfersomal formulations containing different concentration of span 80 (5% to 25%) was determined. The entrapment efficiency of EL-SP1 to EL-SP5 was found to be 23±0.12% to 87±0.32% (Table 1). The maximum entrapment efficiency obtained was 90.4±0.15% for the formulation EL-SP4. The probable reason for higher entrapment efficiency was the presence of ethanol in the transfersomal formulation. Ethanol increases the fluidity and the intralamellar distance of vesicular membranes, which is probably responsible for better entrapment efficiency [14, 18]. The entrapment efficiency in transfersomes depends on the surfactant concentration in the bilayer. Initially, with increasing surfactant concentration, there was an increase in entrapment efficiency. However, after a threshold level (above 20% w/w), a further increase in surfactant concentration led to a decrease in entrapment efficiency. This may be due to the fact that at a certain concentration, surfactant molecule gets associated with the phospholipid bilayer, resulting in better partitioning of drug. So above a 20% concentration of the surfactant, molecules may start forming micelles in a bilayer resulting in pore formation in vesicle membranes and complete conversion of vesicle membranes into mixed micelles. These mixed micelles were reported to have a lower drug carrying capacity and poor skin permeation due to their structural features [19]. The effect of surfactant concentration on the entrapment efficiency of sertraline has been shown diagrammatically in (Fig. 1). From Table 1, it was clear that maximum entrapment efficiency was found in the formulation containing 20% of span 80 (EL-SP4). Thus, EL-SP4 was used to check the maximum drug-loading in the formulation.

### Effect of concentration of drug on entrapment efficiency

**Fig. 2.**
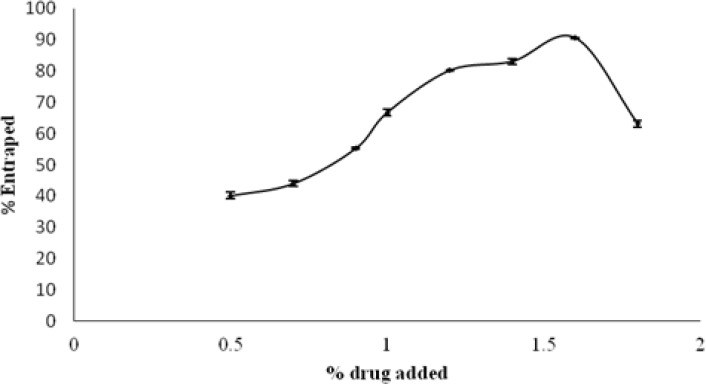
Effect of drug concentration on entrapment efficiency

In the selected transfersomal formulation (EL-SP4), different amounts of the drug in the range of 0.5–1.8% w/w of lecithin was added and all of the drug-loaded transferosomes were examined for maximum entrapment efficiency and for the appearance of drug crystals over a period of 14 days using an optical microscope. Entrapment efficiency was found to first increase with the increase of the drug while no crystal appeared upon addition of 0.5–1.6% drug; this was due to the fact that at first there was an abundance of phosphatidylcholine, and with the increase of the drug, the phosphatidylcholine amount decreased and at the optimum value it showed maximum entrapment. The maximum drug entrapped was found with 1.6% of the drug-loaded formulation (Fig. 2). Loading of the drug above this value gave less entrapment as well as crystals.

### Characterization of transfersomes

#### Vesicle Shape

**Fig. 3.**
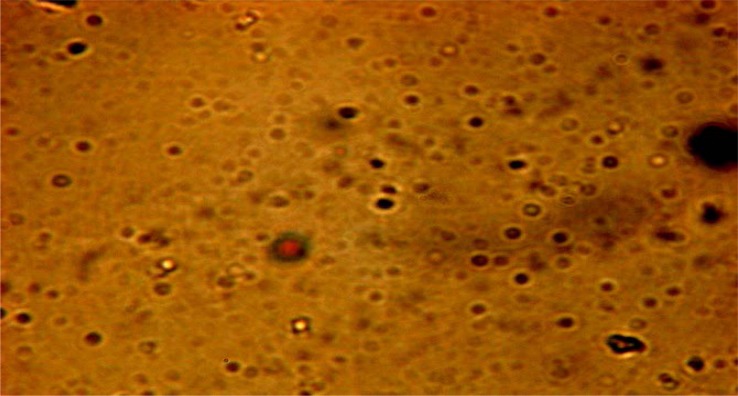
Photomicrograph of EL-SP4

**Fig. 4.**
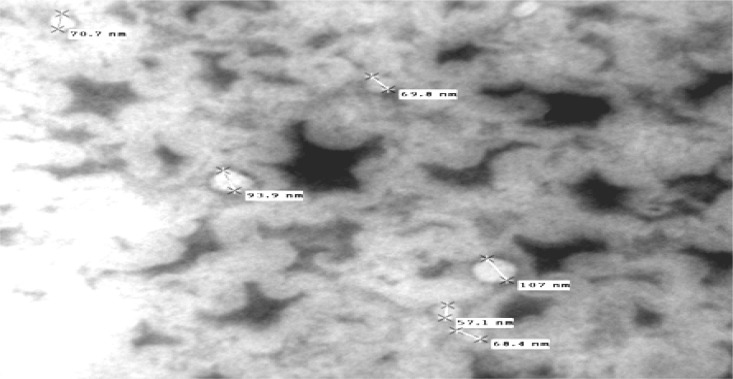
TEM photograph of EL-SP4 formulation

Optical inspection indicated the vesicles appeared as multilamellar vesicles, with the lamellae of vesicles evenly spaced to the core (Fig. 3) and no aggregation irregularities were observed in the system. Results of TEM revealed a positive image in which transfersomes appeared as spherical structures, confirming the vesicular characteristics (Fig. 4).

#### Vesicle size and size distribution

**Fig. 5.**
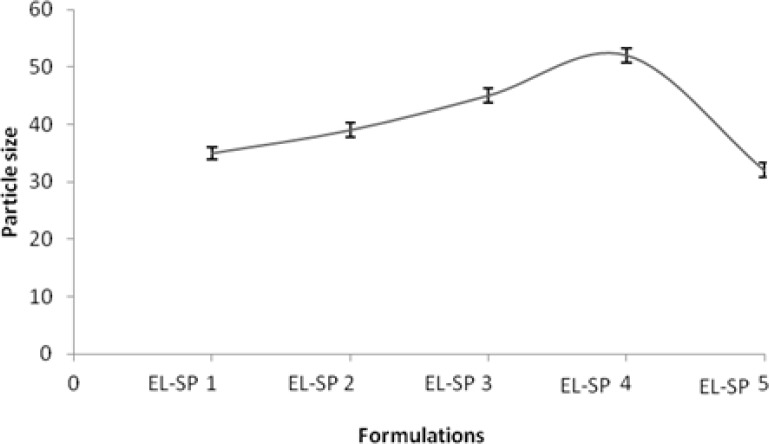
Particle size of different elastic liposomal formulations

Size characterization of transfersomes is essential in ensuring safe and efficient dosages. As the surfactant concentration was kept to a minimum *i.e.* 5%, the vesicle size found to be lowest was 35±0.34 nm (Table 1). When surfactant concentration was increased to 20%, the vesicle size increased substantially and above this concentration it started decreasing (Fig. 5). This was due to the formation of micellar structure instead of vesicles, as discussed earlier [20, 21].

**Fig. 6.**
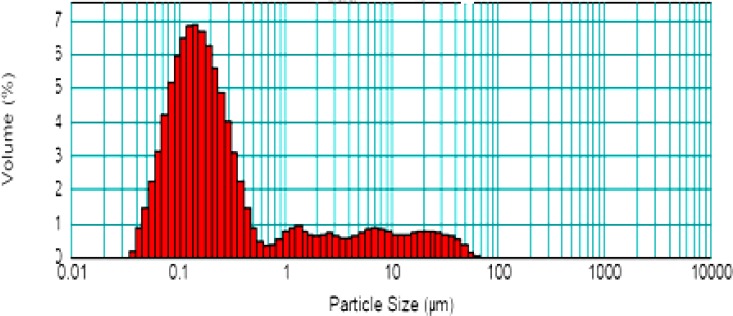
Particle size distribution of EL-SP4

All formulations were found to be in the nano size range with low values of polydispersity index. Polydispersity index is the ratio of the standard deviation to the mean particle size and signifies the uniformity of particle size within the formulation. The polydispersity values of the formulation were found to be low (0.338–0.467), indicating a narrow distribution and uniformity of the particle size within the formulation (Fig. 6).

#### Turbidity measurements

Transformation of transfersomes into mixed micelles is a concentration-dependent process and was governed mainly by the progressive formation of mixed micelles within the bilayer. To support the above fact, turbidity measurements were performed. The formulation EL-SP4 showed maximum turbidity *i.e.* 27.4±0.33 NTU. The results of the turbidity measurement studies (Table 1) sustained the fact that micelles were formed at higher concentrations of surfactant. Thus, it can be concluded that the turbidity of the elastic liposomal formulation increased with an increase in surfactant concentration. The plausible rationale for this situation might be that at low concentrations of surfactant, the partition coefficient favors the lipid phase and caused expansion of the lipid bilayer resulting in increased turbidity of the vesicle dispersion. At the same time, the surfactant also caused fluidization of the bilayer, which is also responsible for the increase in turbidity. After an optimum concentration, conversion of lipid vesicles into mixed micelles began, which have negligible turbidity [14].

#### Drug content determination

The drug content among all of the batches was within the range from 96.56 ± 0.92% to 99.90 ± 0.36% (Table 1). The drug content of the developed formulations was not found to be significantly different (*p* > 0.05, t-test) from the added amount. The results indicate that the process employed to prepare the transfersomes was capable of producing formulations with consistent drug content.

### Viscosity measurement and rheological behaviour of gel

The viscosity of the transfersomal suspension was found to be low (10.25±1.24 to 28.25±0.44 mPaS) and was not suitable for transdermal use, which justified the incorporation of the transfersomal suspension into a gel matrix, resulting in a transfersomal gel having a high value of viscosities. The viscosity of the transfersomal suspension formulation (EL-SP4) (28.25±0.44 mPaS) was significantly increased (p<0.05, t-test) in case of the transfersomal gel (12503.33±0.14 mPaS) due to the incorporation of the carbopol 940 (1.0%) gel matrix, which made the formulation more suitable for the transdermal administration.

**Fig. 7.**
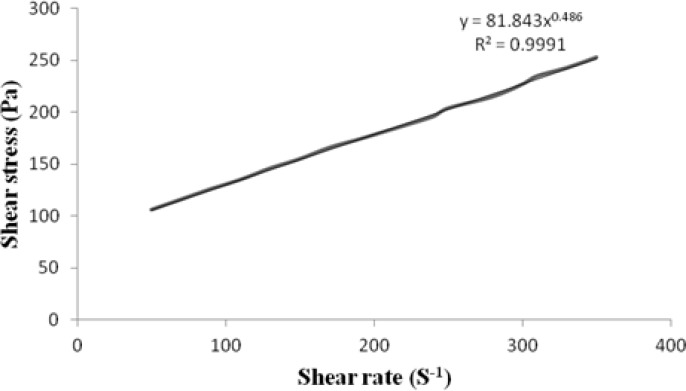
Rheogram of transfersomal gel formulation (EL-SP4).

The rheological behaviour of the gel formulation is governed by its components as well as by the consistency of the formulation. The consistency index of the transfersomal gel formulation (EL-SP4) having high viscosity (12503.33±2.08) was found to be 81.84 Pa.S and had a flow index of (*n*) = 0.486 (Fig. 7). The flow index n is a measure of the deviation of a system from Newtonian behaviour (*n* = 1). A value of *n* < 1 indicates pseudoplastic flow or shear thinning; *n* > 1 indicates dilatant or shear thickening flow. Flow index confers an idea of the flowability of the formulation from the container. The gel showed a flow index of 0.486, indicating pseudoplastic flow behaviour. This pseudoplasticity results from a colloidal network structure that aligns itself in the direction of shear, thereby decreasing the viscosity as the shear rate increases. The pseudoplastic flow performance justifies that the developed system will require some force to expel.

The increase in surfactant concentration leads to an increase in viscosity of the transfersomal gel. Span 80, used as surfactant here, was more soluble in the external aqueous phase. This was due to the concentration of the water-soluble surfactant in the system, which increased the self-association of these amphiphilic molecules and formed different sizes and shapes of micellar aggregates. As the concentration in the external phase increased, the network formed between the surfactant molecules, micelles, and vesicles. The denser the network, the closer the distance between the dispersed phase, and the higher the viscosity.

### In vitro drug release studies through a cellophane membrane

**Fig. 8.**
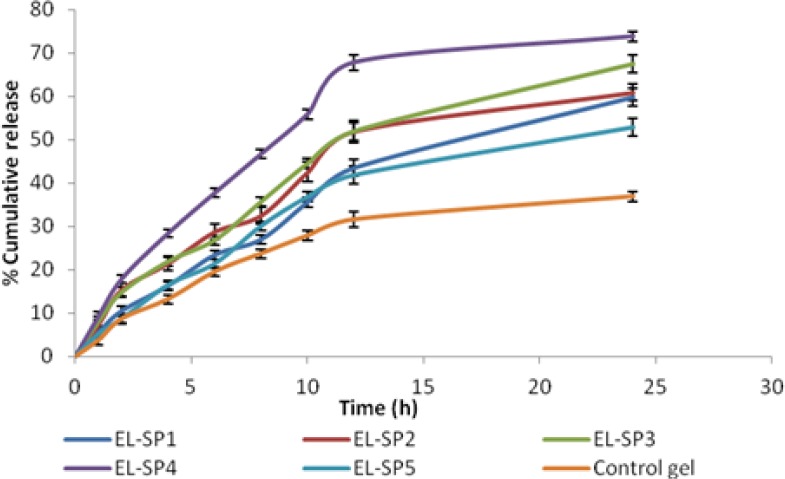
Release profile of sertraline permeated across cellophane membrane from different formulations

Each transfersomal gel formulation and control gel was subjected to *in vitro* drug release studies using a cellophane membrane. The cumulative amount of drug release was calculated for each formulation. Results revealed that the EL-SP4 (formulation with 20% span 80) had the highest cumulative amount of drug release (73%) up to 24 h as compared to other transfersomal gel formulations (52% to 67%). Formulation EL-SP4 was optimized and found to be suitable for further studies. The release rate of sertraline from EL-SP4 was significantly higher (P <0.05, t-test) than the other formulation (Fig. 8). The release experiments clearly indicated sustained-release of sertraline from the transfersomal gel formulation. The maximum release was observed in EL-SP4, because of the higher drug content and entrapment efficiency of the formulation. The maximum release was also due to optimum surfactant concentration (20%), because at this concentration the surfactant molecule gets associated with the phospholipid bilayer resulting in better partitioning of the drug, and resulted in higher drug release from the vesicles.

### Interpretation of release mechanism

The formulation EL-SP4 was best fitted for the Higuchi kinetic equation as the formulation coefficient of correlation values predominates over zero-order and first-order kinetics (Table 2). This indicates the drug permeation mechanism by diffusion, *i.e.* a slow and sustained permeation of the drug from the membrane, as proposed by Higuchi. On the basis of the Korsmeyer-Peppas model, the best fitting was obtained with *n* > 1, indicating super case II. It is well known that when the chain relaxation process is very slow compared with diffusion, the case II transport occurs, which again confirms that the drug release is controlled mainly by diffusion [16].

### Ex vivo skin permeation study

**Fig. 9.**
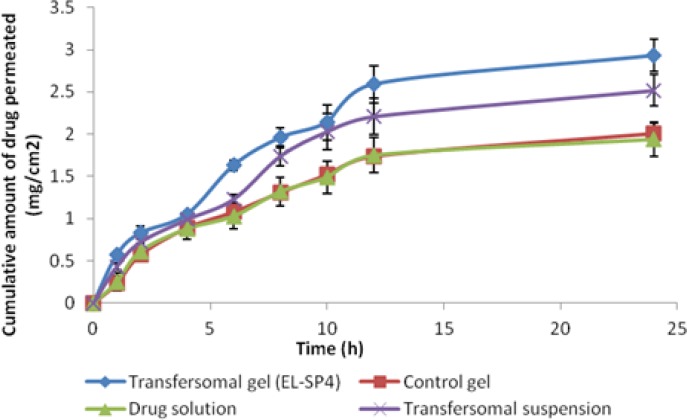
Skin permeation profile of sertraline from various formulations through excised rat skin

Based on the entrapment efficiency, drug content, and *in vitro* release studies, the formulation (EL-SP4) containing 20% of span 80 and 1.6% of the drug was selected for permeation studies through rat skin and compared with the transfersomal suspension, control gel, and drug solution. The *ex vivo* permeation studies provide valuable information about the product behaviour *in vivo* since they indicate the amount of drug available for absorption. The cumulative amount of sertraline permeated per unit area across excised rat skin as the function of time, steady state flux, and lag time were determined. The formulation EL-SP4 and the transfersomal suspension showed the maximum cumulative amount of drug permeate (2.93 ± 0.11 mg/cm^2^) and (2.01 ± 0.15%) (Fig. 9). The transdermal permeation of sertraline from the transfersomal formulation was significantly higher than (p < 0.05, t-test) the control gel and drug solution. The probable reason for the high permeation of the transfersomal gel formulations may be the partitioning of vesicles into the stratum corneum, which is an important process as it drives the partitioning of vesicle-bound drug into the skin. It can, therefore, strongly influence the flux and lag times obtained. One of the reasons for the better skin permeation of the transfersomes was their better partitioning with the stratum corneum and in the deeper layer of skin under the influence of the trans-epidermal osmotic gradient. The transfersomal gel formulation consists of polar lipids (Phospholipid + Surfactant) that have a tendency to attract water due to the energetically favorable interaction between the hydrophilic lipid residues and their proximal water. So, when the transfersomal formulation was applied on the skin surface that was partly dehydrated by water loss due to evaporation, the lipid vesicles felt this osmotic gradient and tried to escape complete drying by moving along this gradient, resulting in the faster partitioning of vesicles into the stratum corneum and other deeper layers of the skin. The above hypothesis was well supported by the report of Kirjavainen *et al*., (1999) that phospholipids affect the stratum corneum lipid bilayer fluidity and improve drug portioning into the bilayer [22].

The *ex vivo* skin permeation of the drug is mainly assessed by its flux, Jss (mg/cm^2^/h). Another important parameter is diffusion lag time (LT) for the drug to reach the receptor compartment. In the present study the transfersomal gel was compared with the transfersomal suspension. The value of the transdermal flux for the EL-SP4 suspension observed was 0.119±2.67 μg/h/cm^2^ and that of the transfersome gel is 0.114±2.57 μg/h/cm^2^. This was about 2.4 times or 2.3 times higher, respectively, than that obtained from the drug solution (0.049±1.27 μg/h/cm^2^). Also, the lag time for the transfersomal suspension and gel was found to be 0.26 h and 0.67 h. It was observed that the flux of the transfersomal gel was lower than the transfersomal suspension, which may be due to the higher viscosity of the formulation. When the flux was compared using the unpaired Student t-test, no significant (p>0.05, t-test) difference was observed (Table 3). Though the transfersomal gel had lower flux, it can be favored over the suspension, due to the prolonged effect and increased viscosity from the viewpoint of its applicability on skin.

### Skin irritation studies

**Fig. 10.**
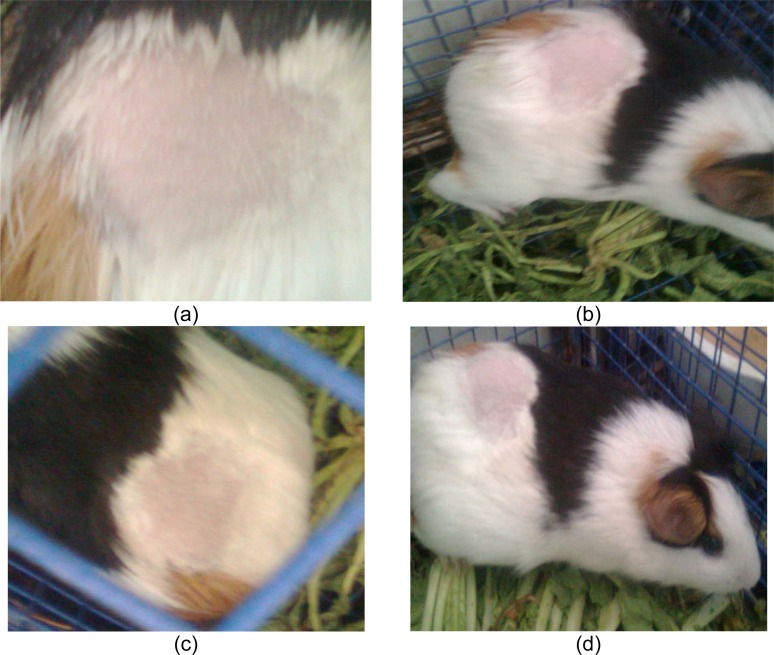
Photograph of the guinea pig (a) before applying any formulation (b)after applying drug solution (c) after applying drug gel (d) after applying transfersomes gel

No erythma and edema were found on the guinea pig’s bare skin after applying the drug gel, transfersomal gel, and plain drug solution. The skin irritation test of the transfersomal gel formulation showed a skin irritation score of zero to one. According to Ammar *et al.,* compounds producing a score of two or less are considered negative (no skin irritation) [15]. Hence, the developed formulation was free of skin irritation (Fig. 10a–d).

### In vivo studies using a modified forced swim model test

**Fig. 11.**
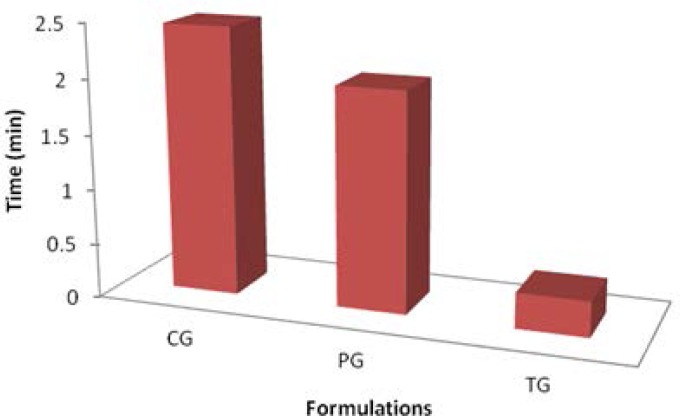
Forced swim model test showing immobility time of mice

It was found that the transfersomal gel (EL-SP4) (0.323 min immobility) showed better antidepressant activity as compared to the control gel (2 min immobility) (Fig.11). This was due to better deformability of the transfersomal gel than of the drug gel. This transfersomal gel formulation increased the struggling behavior of mice by decreasing the time of immobility or depression.

### Stability Studies

**Fig. 12.**
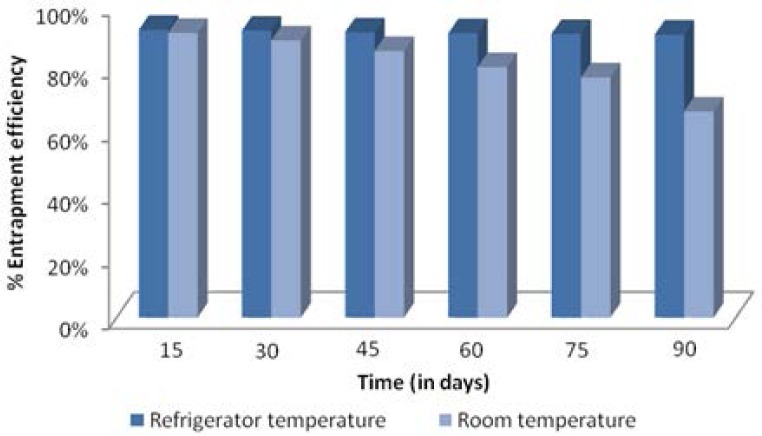
% Entrapment efficiency at refrigerator temperature and room temperature.

**Fig. 13.**
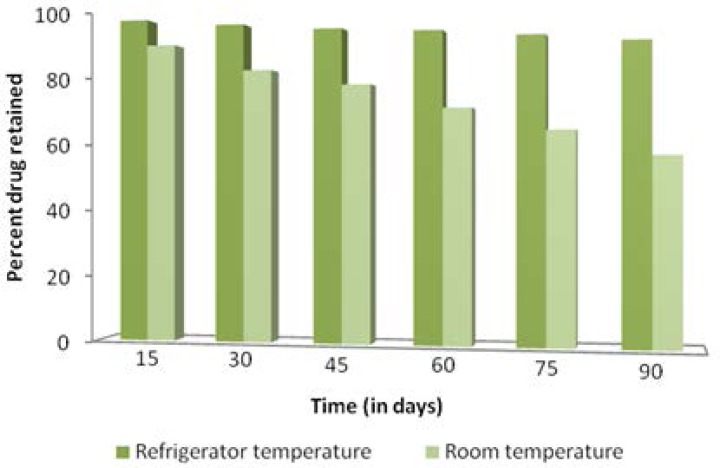
Percent drug retained at refrigerator temperature and room temperature.

The optimized formulation (EL-SP4) was observed in glass vials for a period of 3 months and was visually observed at definitive intervals under an optical microscope for changes in consistency and appearance of the drug crystals. It was found that the consistency of the transfersomal gel did not change after 3 months and no drug crystals appeared even after 3 months both at room temperature (35 ± 2 °C) and refrigerated temperature (4 ± 2 °C). It was also observed that the transfersomes were more stable at refrigerator temperature, as the formulation showed higher entrapment efficiency (> 80%) and percent drug retained (> 90%) than the formulation stored at room temperature, which showed low entrapment efficiency (> 70%) and percent drug retained (> 60%) at the end of 3 months (Fig. 12 and 13).

## Conclusion

The results of the present study indicate that the transfersomal gel formulated by using span 80, soya lecithin, and carbopol (EL-SP4) can be used to enhance skin delivery of sertraline because of excellent release and permeation of the drug. Also, no skin irritation was observed when the gel formulation was applied. The forced swim model test showed that the transfersomal gel formulation has better antidepressant activity because of the increased struggling behaviour of mice and the decreased time of immobility. These studies have shown promising results, hence there is feasibility of delivering sertraline through transfersomal transdermal gel. Thus, the developed transdermal transfersomal formulation may prove to be a promising carrier for sertraline and other drugs, especially due to their simple production and simplistic scale-up.

## Figures and Tables

**Tab. 1.** Characterization of elastic liposomal formulations

**S. No.**	**Formulation code**	**Entrapment efficiency (%)**	**No. of vesicles per mm^3^ 1000 X**	**PDI**	**Drug content (%)**	**Turbidity (NTU)**
1.	EL-SP1	23±0.12	35±0.34	0.442±0.04	97.4±0.34	15.2±0.12
2.	EL-SP2	41±0.14	39±0.23	0.392±0.05	98.5±0.23	19.7±0.32
3.	EL-SP3	79±0.43	45±0.32	0.376±0.02	98.7±0.32	23.1±0.43
4.	EL-SP4	90.4±0.15	52±0.21	0.338±0.08	99.6±0.21	27.4±0.33
5.	EL-SP5	87±0.32	32±0.27	0.467±0.10	97.9±0.27	24.2±0.17

Data are represent of mean ± SD (n=3).

**Tab. 2.** Permeation kinetics of sertraline transfersomal formulations

**Formulation Code**	**Zero order model (r^2^)**	**First order model (r^2^)**	**Higuchi model (r^2^)**	**Korsmeyer-Peppas model (n)**
EL-SP1	0.990	0.977	0.984	1.12
EL-SP2	0.996	0.970	0.983	1.21
EL-SP3	0.986	0.957	0.977	1.18
EL-SP4	0.990	0.907	0.995	1.19
EL-SP5	0.992	0.890	0.985	1.20
Control gel	0.990	0.870	0.991	1.08

**Tab. 3.** Transdermal permeation parameters of different sertraline formulations across rat skin

**Permeation Parameters**	**Transfersomal gel (EL-SP4)**	**Transfersomal suspension**	**Control gel**	**Drug Solution**
Jss^a^(mg/cm^2^/h)	0.114	0.119	0.082	0.049
LT^b^(h)	0.26	0.67	1.23	1.62
ER^c^	1.17	1.32	1.01	1

Data are represent of mean ± SD (n=3).

## References

[1] Kilts CD (2003). Potential New Drug Delivery Systems for Antidepressants: An overview. J Clin Psychiatry.

[2] Kim A, Lee EH, Choi SH, Kim CK (2004). *In vitro* and *in vivo* transfection efficiency of a novel ultra deformable cationic polymer. Biomatetrials.

[3] Vijaya R, Ruckmani K (2011). *In vitro* and *In vivo* characterization of the transdermal delivery of sertraline hydrochloride Films. Daru.

[4] Jain S, Jain P, Umamaheshwari RB, Jain NK (2003). Transfersomes–A novel vesicular carrier for enhanced transdermal delivery: Development, characterization and performance evaluation. Drug Dev Ind Pharm.

[5] Mishra D, Garg M, Dubey V, Jain S, Jain NK (2007). Elastic liposomes mediated transdermal delivery of an anti-hypertensive agent: Propranolol hydrochloride. J Pharm Sci.

[6] Garg T, Jain S, Singh HP, Sharma A, Tiwary AK (2008). Elastic liposomal formulation for sustained delivery of antimigraine drug: in vitro characterization and biological evaluation. Drug Dev Ind Pharm.

[7] Layek B, Mukherjee B (2010). Tamoxifen Citrate Encapsulated Sustained Release Liposomes: Preparation and Evaluation of Physicochemical Properties. Sci Pharm.

[8] El-Maghraby GMM, Williams AC, Barry BW (2001). Skin delivery of 5-Fluorouracil from ultra deformable and traditional liposomes *in vitro*. J Pharm Pharmacol.

[9] Malakar J, Sen SO, Nayak AK, Sen KK (2012). Formulation, optimization and evaluation of transferosomal gel for transdermal insulin delivery. Saudi Pharm J.

[10] Guo J, Ping Q, Sun G, Jiao C (2000). Lecithin vesicular carriers for transdermal delivery of cyclosporine A. Int J Pharm.

[11] Patel MR, Patel RB, Parikh JR, Solanki AB, Patel BG (2009). Effect of formulation components on the in vitro permeation of microemulsion drug delivery system of fluconazole. AAPS PharmSciTech.

[12] Gupta A, Prajapati SK, Balamurugan M, Singh M, Bhatia D (2007). Design and development of a proniosomal transdermal drug delivery system for Captopril. Trop J Pharma Res.

[13] Bachhav YG, Patravale VB (2009). Microemulsion-based vaginal gel of sertraline: Formulation, in vitro evaluation, and stability studies. AAPS PharmSciTech.

[14] Jain S, Jain N, Bhadra D, Tiwary AK, Jain NK (2005). Transdermal delivery of an analgesic agent using elastic liposomes: Preparation, characterization and performance evaluation. Curr Drug Deliv.

[15] Ammar HO, Ghorab M, El-Nahhas SA, Higazy IM (2011). Proniosomes as a carrier system for transdermal delivery of tenoxicam. Int J Pharm.

[16] Bhowmik M, Sanchita D, Chattopadhyay D, Ghosh LK (2011). Study of thermo-sensitive in-situ gels for ocular delivery. Sci Pharm.

[17] Schreier H, Bouwstra JA (1994). Liposomes and niosomes as topical drug carriers: Dermal and transdermal drug delivery. J Control Release.

[18] Touitou E, Dayan N, Bergelson L, Godin B, Eliaz M (2000). Ethosomes- novel vesicular carriers for enhanced delivery: characterization and skin penetration properties. J Control Release.

[19] Lichtenberg D, Robson RJ, Dennis EA (1983). Solublization of phospholipids by detergents: structural and kinetic aspects. Biochim Biophys Acta.

[20] Lasch J, Laub P, Woblrab W (1991). How deep do intact liposomes penetrate into human skin?. J Control Release.

[21] Lopez C, Maza A, Coderch L, Lopez Iglesia C, Wehrli E, Parra JL (1998). Direct formation of mixed micelle in the solublization of phospholipid liposomes by Triton X-100. FEBS Lett.

[22] Kirajavainen M, Monkkonen J, Saukkasaari M, Valjakka-Koskela R, Kiesvaara J (1999). Phospholipids affects stratum corneum lipid bilayer fluidity and drug partitioning into the bilayers. J Control Release.

